# Human Papillomavirus and the DNA Damage Response: Exploiting Host Repair Pathways for Viral Replication

**DOI:** 10.3390/v9080232

**Published:** 2017-08-18

**Authors:** Chelsey C. Spriggs, Laimonis A. Laimins

**Affiliations:** Department of Microbiology-Immunology, Feinberg School of Medicine, Northwestern University, 303 E. Chicago Ave., Chicago, IL 60611, USA; chelsey.spriggs@northwestern.edu

**Keywords:** human papillomavirus, DNA damage response, replication

## Abstract

High-risk human papillomaviruses (HPVs) are the causative agents of cervical and other genital cancers. In addition, HPV infections are associated with the development of many oropharyngeal cancers. HPVs activate and repress a number of host cellular pathways to promote their viral life cycles, including those of the DNA damage response. High-risk HPVs activate the ataxia telangiectasia-mutated (ATM) and ATM and Rad3-related (ATR) DNA damage repair pathways, which are essential for viral replication (particularly differentiation-dependent genome amplification). These DNA repair pathways are critical in maintaining host genomic integrity and stability and are often dysregulated or mutated in human cancers. Understanding how these pathways contribute to HPV replication and transformation may lead to the identification of new therapeutic targets for the treatment of existing HPV infections.

## 1. Introduction

Human papillomaviruses (HPVs) are small, non-enveloped viruses that infect cutaneous and mucosal epithelial cells of the hands, feet, oropharyngeal, and anogenital tracts. Over 200 types of HPV have been identified and about one-third infect the genital tract. Alpha-genus HPVs that infect the genital tract are further classified as either low- or high-risk based on their association with the development of cancers [1,2]. Infections by low-risk HPVs, such as types 6 and 11, are rarely associated with cancers, but often lead to the formation of benign lesions or warts. Approximately ten HPV types, including HPV16, 18, 31, and 45, are considered the etiological agents of cervical cancer, as they are detected in over 99% of cervical carcinomas [3]. These high-risk HPVs have also been linked to the development of anal, vulvar, vaginal, and penile cancers, as well as a rising number of cancers of the oropharyngeal region [4,5].

Approximately 80% of sexually active men and women are infected with HPV at some point during their lifetime, making it one of the most common sexually transmitted viral infections [6]. The majority of HPV infections are cleared by the host’s immune system within two years of initial infection; however, failure to clear this initial infection, in some cases, leads to persistent infection and an increased risk of developing cancer [7]. Cervical cancer was once the leading cause of cancer-related death in women in the United States, but both the incidence and rate of mortality have been reduced with implementation of the Papanicolaou (Pap) smear and the development of prophylactic vaccines [8,9]. Though effective in protecting against initial infection by high-risk types, these vaccines have no therapeutic effect against existing infections. The productive life cycle of these high-risk HPVs is dependent on epithelial differentiation and on activation of host DNA repair pathways that also play critical roles in the development of many cancers. As there currently is no treatment for HPV infection outside of surgery or cryotherapy, understanding the involvement of these pathways in regulating the viral life cycle may lead to new treatment options and further decrease the incidence of HPV-associated carcinomas [10].

## 2. Life Cycle of HPVs

The HPV life cycle is closely linked to the differentiation of host epithelial cells and is regulated by both viral and cellular proteins. In normal squamous epithelia, cells stratify into basal and suprabasal layers. Cellular replication is restricted to the basal layer. Following chromosomal replication and cell division, one of the two daughter cells migrates upward into the suprabasal layers, where it undergoes differentiation and loses its ability to remain active in the cell cycle [11].

Human papillomaviruses infect cells in the basal layer of stratified epithelia that become exposed through microlesions. The initial attachment of HPV to cells is likely mediated through virion binding to heparin sulfate proteoglycans on either the epithelial cell surface or the basement membrane [12,13]. Following entry, virions migrate to the nucleus, where they establish their genomes as extrachromosomal elements, or episomes, at approximately 100 copies per cell [14]. In basal cells, episomes are replicated, along with cellular DNA, during the S phase of the cell cycle with episomal copy numbers maintained at similarly low levels [15].

**Figure 1:**
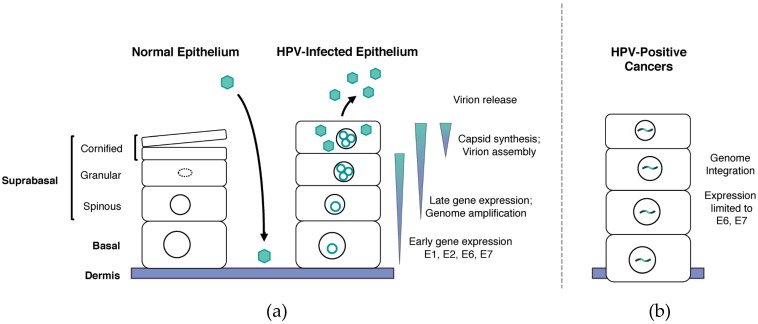
Human papillomavirus (HPV) life cycle. (**a**) Epithelial differentiation program in normal and HPV infected keratinocytes. In normal epithelium, cellular replication occurs in the basal layer of cells. When these cells divide, one daughter cell migrates upwards into differentiated suprabasal layers where it eventually becomes a de-nucleated, keratin filled sac. HPV infects the basal layer of cells, which it gains access to through microlesions in the skin. HPV establishes its DNA as extrachromosomal elements, or episomes, in the nuclei of infected cells. When HPV infected cells divide and migrate into suprabasal layers, cells may bypass regulatory mechanisms to remain active in the cell cycle and amplify their genomes to thousands of copies per cell. Late gene expression and virion assembly as well as release occur in the uppermost layers. (**b**) HPV-positive cancers. In HPV-induced cancers, the viral genome is often found integrated into host DNA. These integration events disrupt E2 regulation of viral gene expression, resulting in increased expression of the E6 and E7 oncoproteins.

As infected cells divide, one daughter cell moves into suprabasal layers. Through the action of viral proteins, a subset of differentiating cells is able to remain active in the cell cycle and progress to G2 phase [16]. In G2, HPV genomes are replicated to thousands of copies per cell in a process termed amplification, and this occurs after cellular replication has been completed [17,18,19]. Concurrent with amplification, capsid synthesis and virion assembly are induced in differentiated cells followed by the release of progeny virions from the epithelial cell surface (Figure 1a).

**Figure 2:**
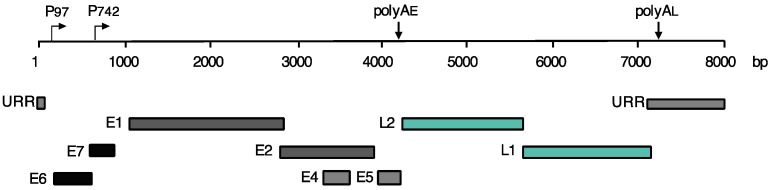
Linear representation of HPV31 DNA. The HPV genome is 8 kb in size and encodes eight viral gene products. The first genes transcribed are the *E6* and *E7* viral oncogenes, followed by the E1 and E2 replication proteins. E4 and E5 are typically expressed during the late stages of viral infection, along with the L1 and L2 capsid proteins. The early (p97) and late (p742) promoters, as well as the corresponding polyadenylation sites (polyA), are designated with arrows. URR: upstream regulatory region.

The circular, double-stranded DNA genomes of HPVs are approximately 8 kb in size and encode for an average of eight open reading frames. Viral genes are divided into two groups, early and late, designated by the stage in the HPV life cycle in which they are predominantly transcribed. In HPV16 and 31, early gene expression is controlled by the p97 promoter, which directs transcripts encoding for the E1 and E2 viral replication proteins as well as the E6 and E7 viral oncoproteins, which are genes necessary for the stable maintenance of viral genomes in basal replicating cells [20,21,22]. The late promoter, designated p742 in HPV31 cells and p670 in HPV16, is activated upon cellular differentiation and controls the expression of genes involved in late viral functions, such as genome amplification and virion production [23,24,25,26]. Late viral gene products include E1, E1^E4, E2, E5, and the L1 and L2 capsid proteins (Figure 2).

In precancerous lesions, viral DNA is maintained as extrachromosomal episomes; but in HPV-induced carcinomas, genomes are frequently found integrated into host chromosomes [27]. Genome integration often disrupts the E2 open reading frame, leading to the loss of E2-mediated transcriptional control and deregulated expression of the *E*6 and *E7* viral oncogenes [28,29]. As such, integration of the viral genome into the host’s DNA is a key step in the progression to malignancy and tumorigenesis (Figure 1b) [30]. Not all HPV-positive cancers have integrated genomes, however, indicating that other mechanisms for enhancing E6 and E7 levels exist.

## 3. The Host DNA Damage Response

**Figure 3:**
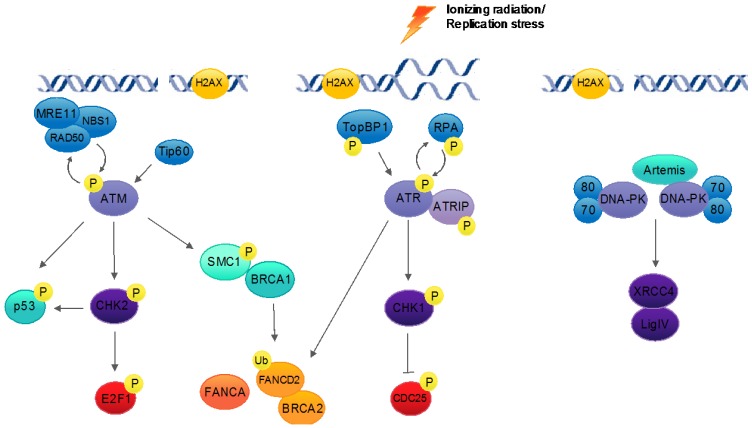
Host DNA damage response pathways. The ataxia telangiectasia-mutated (ATM) and ATM and Rad3-related (ATR) pathways are activated in response to damaged DNA signals, leading to cell cycle checkpoint arrest, DNA repair or apoptosis. ATM is activated by DNA double-strand breaks and initiates downstream response pathways through the phosphorylation of effector proteins, including CHK2 and DNA repair proteins BRCA1 and pSMC1. In addition to the repair of double-strand breaks, ATM is involved in G1/S, intra-S and G2/M cell cycle checkpoint controls, in part through its phosphorylation of p53. ATR responds to replication stress in the form of bulky lesions and the presence of single-stranded DNA at stalled replication forks. It also signals through the activation of specific effector proteins, such as CHK1 and components of the Fanconi Anemia (FA) pathway. The ATM and ATR pathways facilitate DNA repair through the high-fidelity homologous recombination (HR) pathway. DNA double-strand breaks may also be repaired by the DNA-dependent protein kinase (DNA-PK), which coordinates repair through the non-homologous end joining (NHEJ) pathway.

As HPVs encode only two replication proteins—E1 that acts as an origin recognition factor and helicase, and E2 that helps recruit E1 to the viral origin—viral replication relies extensively on host cellular machinery, including those of the DNA damage response [31,32,33]. The DNA damage response is a network of cellular signaling pathways that sense, signal for, and facilitate the repair of damaged DNA. This damage may occur as a result of both environmental and endogenous activity, including ionizing radiation, ultraviolet light, and errors in DNA replication [34]. Mutations in DNA repair genes often lead to genomic instability, congenital disorders and an increased risk of cancer [35]. Central to the DNA damage response are the ataxia telangiectasia-mutated (ATM) and the ATM and Rad3-related (ATR) signaling pathways. ATM and ATR are serine/threonine protein kinases from the phosphatidylinositol 3-kinase-related kinase (PIKK) super family of proteins. They, along with DNA-dependent protein kinase (DNA-PK), are activated in the presence of damaged DNA where they initiate distinct, yet somewhat connected, signaling repair pathways. ATM and DNA-PK are induced in the presence of DNA double-strand breaks, while ATR responds primarily to replication stress in the form of single-stranded DNA at stalled replication forks [35,36] (Figure 3).

DNA double-strand breaks are extremely cytotoxic as they can lead to chromosomal rearrangement, the loss of genetic material and even cell death. These breaks are first recognized by the MRN (MRE11-RAD50-NBS1) complex, which along with the acetyltransferase Tip60, leads to the recruitment and autophosphorylation of ATM at sites of damage. Once activated, ATM phosphorylates a number of downstream proteins, including CHK2 and the histone H2A variant H2AX, to initiate a complex signaling cascade that leads to cell cycle arrest, DNA repair or apoptosis [37]. ATM activates downstream effectors for the repair of double-strand breaks through the high-fidelity homologous recombination pathway in which genetic material is copied and repaired from a homologous sequence, usually from an undamaged sister chromatid. This process is mediated by the *BRCA1* and *BRCA2* breast cancer susceptibility genes, along with the RAD51 and PALB2 DNA repair proteins [38]. ATM pathway activation may also lead to phosphorylation of the tumor suppressor and cell cycle regulatory protein, p53, both directly and indirectly through pCHK2, resulting in cell cycle arrest or apoptosis.

In contrast, DNA-PK promotes the rapid repair of double-strand breaks by the non-homologous end joining (NHEJ) pathway. In this case, double-strand breaks are recognized by the Ku70/80 heterodimer, which acts as a scaffold for the recruitment of additional proteins including the catalytic subunit of DNA-PK (DNA-PKcs). Following end processing by the Artemis endonuclease, DNA-PKcs facilitates the recruitment of the NHEJ ligation complex (XLF-XRCC4-DNA ligase IV) to directly ligate broken DNA ends [37]. Unlike homologous recombination, which is restricted to the S/G2 phases of the cell cycle, NHEJ can occur throughout all phases of the cell cycle, as it does not rely on the presence of a duplicate chromatid for sequence homology. It is, however, more error-prone than homologous recombination and often results in the loss of genetic material.

The ATR kinase responds to a wide range of DNA lesions, including bulky adducts and stalled replication forks. Single-stranded DNA is bound by the replication protein A (RPA), which recruits ATR and its binding partner, ATRIP, to sites of damage. The recruitment of TopBP1 facilitates the phosphorylation and activation of ATR, which in turn phosphorylates its own set of downstream effector proteins, including the CHK1 kinase. This initiates, among other things, cell cycle checkpoint arrest through phosphorylation of the Cdc25 phosphatase [37]. While the ATM and ATR pathways are activated in response to distinct forms of damage, they share several downstream substrates, including proteins of the Fanconi Anemia (FA) pathway [39].

The FA pathway is a DNA damage response pathway that crosstalks with the ATR and ATM pathways in the repair of DNA interstrand crosslinks and cell cycle control. DNA interstrand crosslinks are recognized by the FANCM-FAAP24-MHF complex, which then recruits the large, multi-subunit FA core complex to sites of damage. This results in the ATR-dependent phosphorylation of several FA proteins, including the pathway’s key regulatory protein, FANCD2. These phosphorylation events promote the monoubiquitination of FANCD2 (FANCD2-Ub) by the FA core complex through E3 ubiquitin ligase in its FANCL subunit. FANCD2-Ub localizes with DNA repair proteins, including γ-H2AX and BRCA1, at sites of damage and recruits proteins, such as the FAN1 nuclease, BRCA2, and RAD51 to facilitate DNA repair [40]. FANCD2 is also phosphorylated by the ATM kinase in response to chromosomal double-strand breaks. These phosphorylation events, distinct from those of ATR, lead to an intra-S phase checkpoint response and are not dependent on FANCD2 monoubiquitination [41]. These host DNA repair pathways are intricately involved in the HPV viral life cycle as they are necessary for the efficient replication of viral genomes in infected cells. The role of these pathways in viral replication and how they are regulated by HPV will be discussed in detail below.

## 4. HPV Manipulation of the DNA Damage Response

Several viruses have been shown to modulate components of the DNA damage response for completion of their replicative life cycles [42,43]. For example, adenovirus replication requires inactivation of the MRN complex through its degradation and relocalization, while hepatitis C virus (HCV) inhibits both this complex and the NHEJ pathway during infection [44,45]. In contrast, Epstein-Barr virus (EBV) and human cytomegalovirus (HCMV) activate Tip60, triggering an ATM DNA damage response that is required for efficient lytic replication [46]. HPV both selectively activates and represses members of these pathways to promote viral replication and the completion of its life cycle.

### 4.1. ATM Pathway Activation Is Necessary for Productive Replication

In high-risk HPV-positive cells, the ATM pathway is constitutively activated in the absence of external DNA damaging agents. HPV31, along with 16 and 18, activate an ATM response that is maintained throughout the viral life cycle; and several ATM substrates, including CHK2, NBS1 and BRCA1, are phosphorylated in HPV-positive cells, with phosphorylation levels remaining elevated through differentiation [47,48]. This activation of the ATM pathway is mediated by E7 and additional studies showed that high-level expression of E1 also can activate the ATM response, presumably by inducing the initiation of replication starts from pseudoviral origins located in cellular DNAs that lead to stalled replication forks [47,48,49]. While HPV proteins and replication initiate an ATM-dependent DNA damage response in infected cells, it was unclear whether this activation was a physiological response to the cellular stress of viral replication or an active mechanism taken by the virus to promote its own life cycle.

Recent work indicates this activation is critical for the life cycles of high-risk HPVs. In the presence of high-risk HPV, ATM pathway components accumulate in discrete nuclear foci. These, along with downstream homologous recombination factors, contain replicating viral genomes in both undifferentiated and differentiated cells [50,51]. Inhibitor studies show that while ATM is activated throughout the viral life cycle, it is required for genome amplification only in differentiated cells, but not episomal maintenance in undifferentiated cells, indicating that ATM pathway activation is an essential step in the viral life cycle [47]. Subsequent studies found that along with ATM, many of the pathway’s upstream and downstream components are also required for viral replication, suggesting that the ATM pathway may be activated canonically by HPV during infection.

In normal cells, activation of the ATM pathway requires acetylation of ATM by the Tip60 histone acetyltransferase. Although HPV16 E6 was shown to induce the proteasomal degradation of Tip60, its levels are increased in cells containing the whole viral genome and expressing a wide spectrum of viral proteins [52,53]. In addition, Tip60 is required for ATM activation in HPV-positive cells as well as for genome amplification upon differentiation [53]. In further support of this hypothesis, the expression of both HPV16 and 31 E7 is sufficient to increase the levels of MRN complex components (MRE11, RAD50, and NBS1) that facilitate the recruitment and autophosphorylation of ATM at sites of damage [54]. Following activation, ATM phosphorylates a number of downstream targets, including DNA repair proteins of the homologous recombination pathway. E7 increases the levels of the BRCA1 and RAD51 homologous recombination proteins throughout the viral life cycle and these, along with NBS1, are also required for the amplification of viral genomes in differentiated cells [54,55]. This suggests a possible role for homologous recombination in productive viral replication that may be controlled, in part, by the SIRT1 deacetylase, which regulates the recruitment of both NBS1 and RAD51 to viral genomes [56]. The exact mechanism through which the ATM pathway promotes the amplification of viral genomes in differentiated cells is unclear and remains a major question in the field. One theory is that while basal levels of viral replication are thought to occur through bi-directional (theta) replication, evidence suggests that genomes may instead switch to rolling circle replication and recombination during differentiation-dependent amplification [57]. This rapid induction of viral DNA synthesis may require the ready recruitment of homologous recombination factors by ATM to resolve replicative concatamer intermediates, similar to as seen in simian virus 40 (SV40) [58].

The role of the ATM pathway in HPV replication, however, may be multifaceted as SMC1, a cohesin protein that is phosphorylated by ATM in response to DNA damage signals, is constitutively activated in HPV-positive cells and is also required for genome amplification [59]. pSMC1 localizes to distinct nuclear foci containing γ-H2AX and pCHK2 and binds directly to viral DNA at conserved CTCF sites in HPV late regions [59]. This association of pSMC1 with CTCF, a zinc-finger protein involved in DNA looping and chromatin organization, suggests that in addition to the recruitment of DNA repair factors to viral DNA during recombination, ATM activation may facilitate viral replication, as well as maintenance, by tethering HPV genomes to host chromosomes and allowing for greater access to replication polymerases and machinery [60,61].

### 4.2. ATR Pathway Activation Is Required for Viral Replication

In addition to the ATM pathway, HPV also activates the ATR pathway. Although less is known of its role in the viral life cycle, the transient replication of viral genomes has been shown to induce an ATR-dependent DNA damage response that leads to the accumulation of ATR pathway members, ATRIP and TopBP1, at HPV18 viral replication centers [62]. This induction, which is E1-dependent, suggests that replication stress caused by the initial amplification of viral genomes during the establishment-phase of infection activates an ATR response in cells. Likewise, the stable maintenance of viral episomes in primary keratinocytes leads to ATR pathway activation. Subsequent studies showed that both the total and phosphorylated levels of ATR and its downstream target, CHK1, are increased in cells containing high-risk HPV genomes as well as in cells expressing E6 and E7 alone. Further, the loss of TopBP1 inhibits the phosphorylation ATR and CHK1 and leads to a reduction in the stable maintenance of viral genomes in undifferentiated cells [63]. Importantly, the ATR pathway is also required for productive viral replication, as the inhibition of either pATR or pCHK1 using small molecule inhibitors prevents genome amplification in differentiating cells [63,64].

Overall, these findings suggest that while both ATM and ATR are required for differentiation-dependent amplification, ATR has distinct roles from ATM in the HPV life cycle as it is necessary during all stages of replication. As ATR is often activated in the presence of stalled replication forks, it is possible that HPV activates the ATR pathway to maintain replication fork integrity during the stress of viral replication and to ensure the continued replication of viral genomes.

### 4.3. Inhibition of Downstream Signaling Pathways by E6 and E7

While HPV induces both an ATM and ATR DNA damage response, presumably to gain access to DNA replication and repair machinery for viral replication, this activation also leads to the accumulation of downstream signaling factors involved in cell cycle arrest and apoptosis. As such, several mechanisms are employed by the virus to counteract these anti-proliferative signals and ensure that infected cells remain active in the cell cycle and replicating viral DNA. These events are mediated largely through the coordinated actions of the E6 and E7 viral oncoproteins.

A major target of high-risk E7 is the retinoblastoma (Rb) family of tumor suppressor proteins. Rb, along with p107 and p130, controls the switch between the G1 and S phases of the cell cycle by regulating the activity of E2F transcription factors [65]. The E2F family of proteins controls the transcription of genes involved in S phase replication, cell cycle progression, differentiation, DNA damage repair and apoptosis. Rb represses transcription from E2F-dependent promoters by forming complexes with E2F to prevent its binding at promoter regions. During high-risk HPV infections, E7 directly binds Rb and targets it for degradation by the ubiquitin proteasomal pathway. This leads to the constitutive expression of E2F-responsive genes, including those of the DNA damage response, and allows for viral DNA synthesis [66,67,68,69].

One consequence of E7-induced Rb degradation, as well as ATM/ATR pathway activation by E7 and E1, is an increase in the levels of the tumor suppressor and DNA damage response effector protein, p53, which is involved in regulating the transition from both the G1/S and G2/M phases of the cell cycle [70]. In response to cellular stress, p53 becomes activated, which in turn activates the expression of cell cycle checkpoint control proteins and regulators of apoptosis. To prevent an activated DNA damage response from arresting HPV replicating cells, high-risk E6 proteins bind p53 and recruit the cellular E3 ubiquitin ligase E6-associated protein (E6AP) to induce the its ubiquitination and proteasomal degradation [71,72]. E6 also inhibits p53 by binding to the histone acetyltransferases, p300 and CBP (CREB-binding protein), which are co-activators of p53 activity [73,74]. By preventing its acetylation, these interactions destabilize p53 and further downregulate its activity allowing cells to continue through the cell cycle for viral replication. As p53 is a key regulator of both cell cycle progression and environmental stress responses, its inactivation by E6 also promotes genomic instability in infected cells and increases the risk of cancer.

The potential of high-risk HPVs in progression to malignancy compared to that of the low-risk variety lies mainly in the properties of their E6 and E7 proteins. While there are many functional differences between high- and low-risk E6 and E7, they do share some similarities, such as their regulation of Rb and p53 in infected cells. Although with weaker affinity than their high-risk counterparts, low-risk E7 proteins are capable of binding to, but not degrading, Rb, and their E6 proteins to p53, to prevent the activation of their downstream signaling pathways [75]. Similarly, HPV5 and 8, two beta-genus HPVs that infect cutaneous epithelia, were shown to reduce p53 accumulation in cells through the actions of their E6 proteins. Unlike high-risk types, HPV5 and 8 E6 attenuates p53 activity through interfering with p300-mediated ATR pathway activation. E6 expression reduced ATR mRNA and protein levels, which hindered the downstream phosphorylation and activation of p53 in response to ultraviolet B (UVB)-induced damage [76]. These findings suggest that coordinating the activation and repression of DNA damage response pathway components is a shared, and necessary, step between HPV types for completion of the viral life cycle.

### 4.4. The FA Pathway in Viral Replication and Transformation

Host DNA repair pathways play a complex role in the HPV life cycle and their loss of function may impair viral replication and promote the development of HPV-related cancers. Fanconi Anemia is a genetically heterogeneous disorder caused by a mutation in one or more genes of the FA pathway. FA patients have an increased susceptibility to squamous cell carcinomas (SCCs), particularly those of the oral cavity and anogenital regions, areas that correspond to preferred sites of HPV infection. HPV DNA was detected in over 80% of SCCs from FA patients compared to 36% from control subjects, suggesting that the loss of FA pathway activity may promote viral transformation [77]. FA patients develop these tumors at a relatively young age, which could indicate that genomic instability resulting from the absence of a functional FA pathway decreases the number of additional cellular mutations required for HPV-associated carcinogenesis [78].

Molecular studies found that high-risk HPV E7 upregulates the transcription of FA genes through an Rb-dependent mechanism [69]. Further, high-risk, but not low-risk, E7 is sufficient to activate the FA pathway and stimulates the recruitment of both FANCD2 and BRCA2 to chromatin [79]. The loss of either FANCD2 or core complex component FANCA leads to the post-transcriptional accumulation of E7 and stimulates hyperplastic growth in HPV-positive cells, while E7 expression leads to accelerated chromosomal instability in FA-deficient fibroblasts and an increased susceptibility to head and neck SCCs in FANCD2 knockout mice [80,81,82]. Despite these findings and the correlation between FA loss and HPV-related cancer, the exact role of the FA pathway in the HPV life cycle is still not fully understood.

Recently, FANCD2 was shown to accumulate in distinct nuclear foci, containing BRCA1 and γ-H2AX, that increase upon differentiation [83]. Additionally, FANCD2 localizes to HPV31 viral replication centers and binds to several sites along the viral genome. This binding occurs in both undifferentiated and differentiated cells, but decreases upon differentiation suggesting that the FA pathway may be involved primarily in the replication of viral genomes in undifferentiated cells [83]. In line with this, the loss of FANCD2 led to reduced episomal maintenance in undifferentiated cells, which may ultimately promote the integration of viral genomes into host DNA and could explain the increased susceptibility to HPV-positive cancers in these patients [83]. Another study reported that the FA pathway limits viral replication, as the knockdown of FANCD2 stimulated genome amplification in differentiating cells [80]. This may be the result of the enhanced generation of multimeric genomes in differentiating cells, but further studies will need to be conducted to resolve these differing models of HPV activation in FA-deficient cells.

Activation of the FA pathway is observed similarly in other DNA viruses, including herpes simplex virus-1 (HSV-1), adenovirus and SV40, where it is also important for productive replication and growth [84,85,86]. In HSV-1, this was partially attributed to the role of the FA pathway in promoting homologous recombination over NHEJ repair. Although little is known of the role of DNA-PK and the NHEJ pathway in the HPV life cycle, the FA pathway is likely involved in the choice between these DNA repair mechanisms as FANCD2-Ub mediates the recruitment of homologous recombination factors, including BRCA1, BRCA2, and PALB2, to sites of damage and is required for RAD51 focus formation [87,88,89]. Moreover, defects in either FANCD2 or FANCJ stimulates the phosphorylation of the NHEJ DNA-PKcs kinase. FA-deficient cells exhibit higher rates of microdeletions and insertions, which is often indicative of hyperactive NHEJ repair [90,91]. It is possible that HPV activates the FA pathway, either directly or indirectly through the ATR pathway, and recruits FANCD2 to the viral genome in order to bring downstream homologous recombination proteins to viral DNA during replication. ATM also activates a FANCD2-dependent intra-S phase cell cycle checkpoint, although its significance in HPV life cycle has not yet been studied in depth. As HPV E6 and E7 utilize several mechanisms to ensure cell cycle progression in the presence of DNA damage signals, it is possible that they also inhibit activation of FANCD2 in this context, which would indicate a more complicated relationship between HPV and the FA pathway than previously suggested.

## 5. Discussion

Human papillomaviruses have adapted several mechanisms to ensure the completion of their viral life cycles, including the use of host cellular machinery to replicate their genomes. The host DNA damage response is a network of signaling and repair pathways that safeguard the cellular genome from insult and prevent the propagation of damaged DNA. Infection by HPV, as well as many other viruses, is sufficient to activate these DNA damage repair pathways, which can be both advantageous and detrimental to the viral life cycle. These pathways are involved in the faithful replication of cellular DNA and HPV has evolved to exploit these activities for viral replication.

Studies showing that HPV activates both the ATM and ATR pathways, as well as the FA pathway, for viral replication have led to suggestions that components of these pathways could serve as promising therapeutic targets for the treatment of HPV infections. As there is currently no treatment for HPV infection outside of surgical intervention or cryotherapy, developing better therapeutics is a research priority in the field. DNA damage response pathways are essential for maintaining genomic stability in cells, and their dysregulation or loss is a key step in carcinogenesis. Defects in components of these pathways, often give cancer cells survival and proliferative advantages, but researchers believe that it may also drive a dependence on other parallel pathways that can then be exploited for treatment [92]. This same idea could be applied in the development of therapeutics against HPV infection. Using specific ATM and ATR inhibitors to target viral replication, particularly genome amplification in differentiated cells, as the loss of FA-dependent episomal maintenance could lead to increased rates of integration, may reduce the cells ability to support successful infection.

Studying the relationship between HPV and the DNA damage response may prove especially useful in the control of HPV-associated cancers of the oropharyngeal region, which are rising at epidemic proportion [93]. Although there is currently no effective screening strategy for non-genital HPV-associated cancers, there are links between these cancers and the DNA damage response that should be explored further. FA patients have an increased incidence of HPV-related cancer in the head and neck region and deficiencies in the FA pathway increased the sensitivity to HPV-associated head and neck cancer in E7 transgenic mice, suggesting that an intact FA pathway may limit viral transformation in this region [77,82]. Investigating the mechanisms underlying this inhibitory effect may provide important insights that could be helpful in the future treatment and prevention of these cancers.

Over the past several years, the involvement of DNA damage response pathways in HPV replication has become increasingly clear; however, the extent to which this complex network regulates the HPV viral life cycle is still not fully understood. In all, continued research into the roles that these pathways play in viral replication and transformation is critical, and may lead a better understanding of HPV itself and of DNA damage response pathways in cancer as a whole.

## References

[1] Doorbar J., Quint W., Banks L., Bravo I.G., Stoler M., Broker T.R., Stanley M.A. (2012). The biology and life-cycle of human papillomaviruses. Vaccine.

[2] Bernard H.U., Burk R.D., Chen Z., van Doorslaer K., Zur Hausen H., de Villiers E.M. (2010). Classification of papillomaviruses (PVs) based on 189 PV types and proposal of taxonomic amendments. Virology.

[3] Walboomers J.M., Jacobs M.V., Manos M.M., Bosch F.X., Kummer J.A., Shah K.V., Snijders P.J., Peto J., Meijer C.J., Muñoz N. (1999). Human papillomavirus is a necessary cause of invasive cervical cancer worldwide. J. Pathol..

[4] Munger K. (2002). The role of human papillomaviruses in human cancers. Front. Biosci..

[5] Viens L.J., Henley S.J., Watson M., Markowitz L.E., Thomas C.C., Thompson T.D., Razzaghi H., Saraiya M. (2016). Human papillomavirus-associated cancers-United States, 2008–2012. MMWR Morb. Mortal. Wkly. Rep..

[6] Myers E.R., McCrory D.C., Nanda K., Bastian L., Matchar D.B. (2000). Mathematical model for the natural history of human papillomavirus infection and cervical carcinogenesis. Am. J. Epidemiol..

[7] Zur Hausen H. (2002). Papillomaviruses and cancer: From basic studies to clinical application. Nat. Rev. Cancer.

[8] Saraiya M., Steben M., Watson M., Markowitz L. (2013). Evolution of cervical cancer screening and prevention in United States and Canada: Implications for public health practitioners and clinicians. Prev. Med..

[9] Torre L.A., Bray F., Siegel R.L., Ferlay J., Lortet-Tieulent J., Jemal A. (2015). Global cancer statistics, 2012. CA Cancer J. Clin..

[10] Wright T.C., Massad L.S., Dunton C.J., Spitzer M., Wilkinson E.J., Solomon D., American Society for Colposcopy, Cervical Pathology-sponsored Consensus Conference (2007). 2006 consensus guidelines for the management of women with cervical intraepithelial neoplasia or adenocarcinoma in situ. Am. J. Obstet. Gynecol..

[11] Fuchs E. (1994). Epidermal differentiation and keratin gene expression. Princess Takamatsu Symp..

[12] Johnson K.M., Kines R.C., Roberts J.N., Lowy D.R., Schiller J.T., Day P.M. (2009). Role of heparan sulfate in attachment to and infection of the murine female genital tract by human papillomavirus. J. Virol..

[13] Kines R.C., Thompson C.D., Lowy D.R., Schiller J.T., Day P.M. (2009). The initial steps leading to papillomavirus infection occur on the basement membrane prior to cell surface binding. Proc. Natl. Acad. Sci. USA.

[14] Stubenrauch F., Laimins L.A. (1999). Human papillomavirus life cycle: Active and latent phases. Semin. Cancer Biol..

[15] Lambert P.F. (1991). Papillomavirus DNA replication. J. Virol..

[16] Cheng S., Schmidt-Grimminger D.C., Murant T., Broker T.R., Chow L.T. (1995). Differentiation-dependent up-regulation of the human papillomavirus E7 gene reactivates cellular DNA replication in suprabasal differentiated keratinocytes. Genes Dev..

[17] Sakakibara N., Chen D., McBride A.A. (2013). Papillomaviruses use recombination-dependent replication to vegetatively amplify their genomes in differentiated cells. PLoS Pathog..

[18] Lee C., Laimins L.A., Dimaio D., Garcea D. (2007). The differentiation-dependent life cycle of human papillomaviruses in keratinocytes. The Papillomaviruses.

[19] Wang H.K., Duffy A.A., Broker T.R., Chow L.T. (2009). Robust production and passaging of infectious HPV in squamous epithelium of primary human keratinocytes. Genes Dev..

[20] Rohlfs M., Winkenbach S., Meyer S., Rupp T., Durst M. (1991). Viral transcription in human keratinocyte cell lines immortalized by human papillomavirus type-16. Virology.

[21] Del Vecchio A.M., Romanczuk H., Howley P.M., Baker C.C. (1992). Transient replication of human papillomavirus DNAs. J. Virol..

[22] Ozbun M.A. (2002). Human papillomavirus type 31b infection of human keratinocytes and the onset of early transcription. J. Virol..

[23] Hummel M., Hudson J.B., Laimins L.A. (1992). Differentiation-induced and constitutive transcription of human papillomavirus type 31b in cell lines containing viral episomes. J. Virol..

[24] Wilson R., Ryan G.B., Knight G.L., Laimins L.A., Roberts S. (2007). The full-length E1^E4 protein of human papillomavirus type 18 modulates differentiation-dependent viral DNA amplification and late gene expression. Virology.

[25] Fehrmann F., Klumpp D.J., Laimins L.A. (2003). Human papillomavirus type 31 E5 protein supports cell cycle progression and activates late viral functions upon epithelial differentiation. J. Virol..

[26] Genther S.M., Sterling S., Duensing S., Munger K., Sattler C., Lambert P.F. (2003). Quantitative role of the human papillomavirus type 16 E5 gene during the productive stage of the viral life cycle. J. Virol..

[27] Moody C.A., Laimins L.A. (2010). Human papillomavirus oncoproteins: Pathways to transformation. Nat. Rev. Cancer.

[28] Schwarz E., Freese U.K., Gissmann L., Mayer W., Roggenbuck B., Stremlau A., zur Hausen H. (1985). Structure and transcription of human papillomavirus sequences in cervical carcinoma cells. Nature.

[29] Baker C.C., Phelps W.C., Lindgren V., Braun M.J., Gonda M.A., Howley P.M. (1987). Structural and transcriptional analysis of human papillomavirus type 16 sequences in cervical carcinoma cell lines. J. Virol..

[30] Jeon S., Allen-Hoffmann B.L., Lambert P.F. (1995). Integration of human papillomavirus type 16 into the human genome correlates with a selective growth advantage of cells. J. Virol..

[31] Frattini M.G., Laimins L.A. (1994). Binding of the human papillomavirus E1 origin-recognition protein is regulated through complex formation with the E2 enhancer-binding protein. Proc. Natl Acad. Sci. USA.

[32] Sedman J., Stenlund A. (1995). Co-operative interaction between the initiator E1 and the transcriptional activator E2 is required for replicator specific DNA replication of bovine papillomavirus in vivo and in vitro. EMBO J..

[33] Mohr I.J., Clark R., Sun S., Androphy E.J., MacPherson P., Botchan M.R. (1990). Targeting the E1 replication protein to the papillomavirus origin of replication by complex formation with the E2 transactivator. Science.

[34] Ciccia A., Elledge S.J. (2010). The DNA damage response: Making it safe to play with knives. Mol. Cell.

[35] Eyfjord J.E., Bodvarsdottir S.K. (2005). Genomic instability and cancer: Networks involved in response to DNA damage. Mutat. Res..

[36] Sulli G., Di Micco R., d’Adda di Fagagna F. (2012). Crosstalk between chromatin state and DNA damage response in cellular senescence and cancer. Nat. Rev. Cancer.

[37] Hollingworth R., Grand R.J. (2015). Modulation of DNA damage and repair pathways by human tumour viruses. Viruses.

[38] Sy S.M., Huen M.S., Chen J. (2009). PALB2 is an integral component of the BRCA complex required for homologous recombination repair. Proc. Natl Acad. Sci. USA.

[39] Grompe M. (2002). FANCD2: A branch-point in DNA damage response?. Nat. Med..

[40] Kee Y., D’Andrea A.D. (2010). Expanded roles of the Fanconi Anemia pathway in preserving genomic stability. Genes Dev..

[41] Taniguchi T., Garcia-Higuera I., Xu B., Andreassen P.R., Gregory R.C., Kim S.T., Lane W.S., Kastan M.B., D’Andrea A.D. (2002). Convergence of the Fanconi Anemia and ataxia telangiectasia signaling pathways. Cell.

[42] Wallace N.A., Galloway D.A. (2014). Manipulation of cellular DNA damage repair machinery facilitates propagation of human papillomaviruses. Semin. Cancer Biol..

[43] Turnell A.S., Grand R.J. (2012). DNA viruses and the cellular DNA-damage response. J. Gen. Virol..

[44] Stracker T.H., Carson C.T., Weitzman M.D. (2002). Adenovirus oncoproteins inactivate the Mre11-Rad50-NBS1 DNA repair complex. Nature.

[45] Machida K., McNamara G., Cheng K.T., Huang J., Wang C.H., Comai L., Ou J.H., Lai M.M. (2010). Hepatitis C virus inhibits DNA damage repair through reactive oxygen and nitrogen species and by interfering with the ATM-NBS1/Mre11/Rad50 DNA repair pathway in monocytes and hepatocytes. J. Immunol..

[46] Li R., Zhu J., Xie Z., Liao G., Liu J., Chen M.R., Hu S., Woodard C., Lin J., Taverna S.D. (2011). Conserved herpesvirus kinases target the DNA damage response pathway and Tip60 histone acetyltransferase to promote virus replication. Cell Host Microbe.

[47] Moody C.A., Laimins L.A. (2009). Human papillomaviruses activate the ATM DNA damage pathway for viral genome amplification upon differentiation. PLoS Pathog..

[48] Sakakibara N., Mitra R., McBride A.A. (2011). The papillomavirus E1 helicase activates a cellular DNA damage response in viral replication foci. J. Virol..

[49] Kadaja M., Isok-Paas H., Laos T., Ustav E., Ustav M. (2009). Mechanism of genomic instability in cells infected with the high-risk human papillomaviruses. PLoS Pathog..

[50] Gillespie K.A., Mehta K.P., Laimins L.A., Moody C.A. (2012). Human papillomaviruses recruit cellular DNA repair and homologous recombination factors to viral replication centers. J. Virol..

[51] McKinney C.C., Hussmann K.L., McBride A.A. (2015). The role of the DNA damage response throughout the papillomavirus life cycle. Viruses.

[52] Jha S., Vande Pol S., Banerjee N.S., Dutta A.B., Chow L.T., Dutta A. (2010). Destabilization of Tip60 by human papillomavirus E6 results in attenuation of Tip60-dependent transcriptional regulation and apoptotic pathway. Mol. Cell.

[53] Hong S., Dutta A., Laimins L.A. (2015). The acetyltransferase Tip60 is a critical regulator of the differentiation-dependent amplification of human papillomaviruses. J. Virol..

[54] Anacker D.C., Gautam D., Gillespie K.A., Chappell W.H., Moody C.A. (2014). Productive replication of human papillomavirus 31 requires DNA repair factor NBS1. J. Virol..

[55] Chappell W.H., Gautam D., Ok S.T., Johnson B.A., Anacker D.C., Moody C.A. (2015). Homologous recombination repair factors Rad51 and BRCA1 are necessary for productive replication of human papillomavirus 31. J. Virol..

[56] Langsfeld E.S., Bodily J.M., Laimins L.A. (2015). The deacetylase sirtuin 1 regulates human papillomavirus replication by modulating histone acetylation and recruitment of DNA damage factors NBS1 and Rad51 to viral genomes. PLoS Pathog..

[57] Flores E.R., Lambert P.F. (1997). Evidence for a switch in the mode of human papillomavirus type 16 DNA replication during the viral life cycle. J. Virol..

[58] Sowd G.A., Mody D., Eggold J., Cortez D., Friedman K.L., Fanning E. (2014). SV40 utilizes ATM kinase activity to prevent non-homologous end joining of broken viral DNA replication products. PLoS Pathog..

[59] Mehta K., Gunasekharan V., Satsuka A., Laimins L.A. (2015). Human papillomaviruses activate and recruit SMC1 cohesin proteins for the differentiation-dependent life cycle through association with CTCF insulators. PLoS Pathog..

[60] Sun M., Nishino T., Marko J.F. (2013). The SMC1–SMC3 cohesin heterodimer structures DNA through supercoiling-dependent loop formation. Nucleic Acids Res..

[61] Feeney K.M., Wasson C.W., Parish J.L. (2010). Cohesin: A regulator of genome integrity and gene expression. Biochem. J..

[62] Reinson T., Toots M., Kadaja M., Pipitch R., Allik M., Ustav E., Ustav M. (2013). Engagement of the ATR-dependent DNA damage response at the human papillomavirus 18 replication centers during the initial amplification. J. Virol..

[63] Hong S., Cheng S., Iovane A., Laimins L.A. (2015). STAT-5 regulates transcription of the topoisomerase II beta-binding protein 1 (TopBP1) gene to activate the ATR pathway and promote human papillomavirus replication. Am. Soc. Microbiol..

[64] Edwards T.G., Helmus M.J., Koeller K., Bashkin J.K., Fisher C. (2013). Human papillomavirus episome stability is reduced by aphidicolin and controlled by DNA damage response pathways. J. Virol..

[65] Dyson N. (1998). The regulation of E2F by pRB-family proteins. Genes Dev..

[66] Munger K., Werness B.A., Dyson N., Phelps W.C., Harlow E., Howley P.M. (1989). Complex formation of human papillomavirus E7 proteins with the retinoblastoma tumor suppressor gene product. EMBO J..

[67] Boyer S.N., Wazer D.E., Band V. (1996). E7 protein of human papilloma virus-16 induces degradation of retinoblastoma protein through the ubiquitin-proteasome pathway. Cancer Res..

[68] Chellappan S., Kraus V.B., Kroger B., Munger K., Howley P.M., Phelps W.C., Nevins J.R. (1992). Adenovirus E1A, simian virus 40 tumor antigen, and human papillomavirus E7 protein share the capacity to disrupt the interaction between transcription factor E2F and the retinoblastoma gene product. Proc. Natl. Acad. Sci. USA.

[69] Hoskins E.E., Gunawardena R.W., Habash K.B., Wise-Draper T.M., Jansen M., Knudsen E.S., Wells S.I. (2008). Coordinate regulation of Fanconi Anemia gene expression occurs through the RB/E2F pathway. Oncogene.

[70] Jones D.L., Thompson D.A., Munger K. (1997). Destabilization of the RB tumor suppressor protein and stabilization of p53 contribute to HPV type 16 E7-induced apoptosis. Virology.

[71] Scheffner M., Huibregtse J.M., Vierstra R.D., Howley P.M. (1993). The HPV-16 E6 and E6-AP complex functions as a ubiquitin-protein ligase in the ubiquitination of p53. Cell.

[72] Scheffner M., Werness B.A., Huibregtse J.M., Levine A.J., Howley P.M. (1990). The E6 oncoprotein encoded by human papillomavirus types 16 and 18 promotes the degradation of p53. Cell.

[73] Patel D., Huang S.M., Baglia L.A., McCance D.J. (1999). The E6 protein of human papillomavirus type 16 binds to and inhibits co-activation by CBP and p300. EMBO J..

[74] Zimmermann H., Degenkolbe R., Bernard H.U., O’Connor M.J. (1999). The human papillomavirus type 16 E6 oncoprotein can down-regulate p53 activity by targeting the transcriptional coactivator CBP/p300. J. Virol..

[75] Klingelhutz A.J., Roman A. (2012). Cellular transformation by human papillomaviruses: Lessons learned by comparing high- and low-risk viruses. Virology.

[76] Wallace N.A., Robinson K., Howie H.L., Galloway D.A. (2012). HPV 5 and 8 E6 abrogate ATR activity resulting in increased persistence of UVB induced DNA damage. PLoS Pathog..

[77] Kutler D.I., Wreesmann V.B., Goberdhan A., Ben-Porat L., Satagopan J., Ngai I., Huvos A.G., Giampietro P., Levran O., Pujara K. (2003). Human papillomavirus DNA and p53 polymorphisms in squamous cell carcinomas from Fanconi Anemia patients. J. Natl. Cancer Inst..

[78] Lowy D.R., Gillison M.L. (2003). A new link between Fanconi Anemia and human papillomavirus-associated malignancies. J. Natl. Cancer Inst..

[79] Spardy N., Duensing A., Charles D., Haines N., Nakahara T., Lambert P.F., Duensing S. (2007). The human papillomavirus type 16 E7 oncoprotein activates the Fanconi Anemia (FA) pathway and causes accelerated chromosomal instability in FA cells. J. Virol..

[80] Hoskins E.E., Morreale R.J., Werner S.P., Higginbotham J.M., Laimins L.A., Lambert P.F., Brown D.R., Gillison M.L., Nuovo G.J., Witte D.P. (2012). The Fanconi Anemia pathway limits human papillomavirus replication. J. Virol..

[81] Hoskins E.E., Morris T.A., Higginbotham J.M., Spardy N., Cha E., Kelly P., Williams D.A., Wikenheiser-Brokamp K.A., Duensing S., Wells S.I. (2009). Fanconi Anemia deficiency stimulates HPV-associated hyperplastic growth in organotypic epithelial raft culture. Oncogene.

[82] Park J.W., Pitot H.C., Strati K., Spardy N., Duensing S., Grompe M., Lambert P.F. (2010). Deficiencies in the Fanconi Anemia DNA damage response pathway increase sensitivity to HPV-associated head and neck cancer. Cancer Res..

[83] Spriggs C.C., Laimins L.A. (2017). FANCD2 binds human papillomavirus genomes and associates with a distinct set of DNA repair proteins to regulate viral replication. Microbiology.

[84] Karttunen H., Savas J.N., McKinney C., Chen Y.H., Yates J.R., Hukkanen V., Huang T.T., Mohr I. (2014). Co-opting the Fanconi Anemia genomic stability pathway enables herpesvirus DNA synthesis and productive growth. Mol. Cell.

[85] Cherubini G., Naim V., Caruso P., Burla R., Bogliolo M., Cundari E., Benihoud K., Saggio I., Rosselli F. (2011). The FANC pathway is activated by adenovirus infection and promotes viral replication-dependent recombination. Nucleic Acids Res..

[86] Boichuk S., Hu L., Hein J., Gjoerup O.V. (2010). Multiple DNA damage signaling and repair pathways deregulated by simian virus 40 large t antigen. J. Virol..

[87] Taniguchi T., Garcia-Higuera I., Andreassen P.R., Gregory R.C., Grompe M., D’Andrea A.D. (2002). S-phase-specific interaction of the Fanconi anemia protein, FANCD2, with BRCA1 and Rad51. Blood.

[88] Wang X., Andreassen P.R., D’Andrea A.D. (2004). Functional interaction of monoubiquitinated FANCD2 and BRCA2/FANCD1 in chromatin. Mol. Cell. Biol..

[89] Xia B., Dorsman J.C., Ameziane N., de Vries Y., Rooimans M.A., Sheng Q., Pals G., Errami A., Gluckman E., Llera J. (2007). Fanconi Anemia is associated with a defect in the BRCA2 partner PALB2. Nat. Genet..

[90] Moldovan G.L., D’Andrea A.D. (2009). How the Fanconi Anemia pathway guards the genome. Ann. Rev. Genet..

[91] Romick-Rosendale L.E., Hoskins E.E., Privette Vinnedge L.M., Foglesong G.D., Brusadelli M.G., Potter S.S., Komurov K., Brugmann S.A., Lambert P.F., Kimple R.J. (2016). Defects in the Fanconi Anemia pathway in head and neck cancer cells stimulate tumor cell invasion through DNA-PK and Rac1 signaling. Clin. Cancer Res..

[92] Weber A.M., Ryan A.J. (2015). ATM and ATR as therapeutic targets in cancer. Pharmacol. Ther..

[93] Mallen-St Clair J., Alani M., Wang M.B., Srivatsan E.S. (2016). Human papillomavirus in oropharyngeal cancer: The changing face of a disease. Biochim. Biophys. Acta.

